# Social Anxiety, Social Support, and Quality of Life in Patients With Epilepsy at a Tertiary Care Hospital in Saudi Arabia

**DOI:** 10.7759/cureus.45447

**Published:** 2023-09-18

**Authors:** Faisal I Alkoblan, Musaeb M Alsoadan, Abdulrahman A Alhajri, Mohammed M Almousa, Faisal S Alsalamah, Ambreen Kazi, Fahad A Bashiri, Bandar Aljafen

**Affiliations:** 1 College of Medicine, King Saud University, Riyadh, SAU; 2 Princess Nora Bent Abdullah Research Chair for Women's Health Research, Deanship of Scientific Research, King Saud University, Riyadh, SAU; 3 Department of Family and Community Medicine, King Saud University Medical City, Riyadh, SAU; 4 Division of Pediatric Neurology, Department of Pediatrics, College of Medicine, King Saud University Medical City, King Saud University, Riyadh, SAU; 5 Division of Neurology, College of Medicine, King Saud University Medical City, King Saud University, Riyadh, SAU; 6 College of Medicine, Dar Al Uloom University, Riyadh, SAU

**Keywords:** saudi arabia, quality of life (qol), social anxiety, social support, epilepsy

## Abstract

Objectives

The objectives of this study were to measure the prevalence of social anxiety disorder and its association with quality of life (QoL) in patients with epilepsy (PWE) in King Khalid University Hospital, Riyadh City, Saudi Arabia.

Methodology

A self-administrated, online, questionnaire-based, cross-sectional study was conducted at King Khalid University Hospital, Riyadh, Kingdom of Saudi Arabia on PWE 18 years of age and above from February 2021 to January 2022. Social anxiety and the five domains of QoL were measured using the validated Arabic versions of the Liebowitz social anxiety scale and European quality-of-life 5-dimensions 3-levels (EQ-5D-3L), respectively. Information was collected on sociodemographic characteristics, social support, and adverse life events.

Results

This study included 246 patients, of which approximately 25% and 15% had mild/moderate and severe/very severe levels of social anxiety, respectively. Severe social anxiety was significantly associated with poor QoL domains, namely, restricted mobility (2.65 [1.00, 6.99]), inability to perform usual activities (3.88 [1.61, 9.36]), pain or discomfort (3.21 [1.38, 7.48]), and anxiety and depression (5.77 [2.45, 13.61]). Similarly, the lack of social support was also significantly associated with poor QoL, such as restricted mobility (2.42 [1.12, 5.22]), restricted self-care (3.64 [1.18, 11.17]), inability to perform usual activities (2.86 [1.42, 5.75]), pain/discomfort (2.53 [1.38, 4.66]), and anxiety and depression (1.93 [1.04, 3.57]). Females showed higher odds for restricted mobility (2.79 [1.29, 6.03]) and low education with limited self-care (7.38 [1.49, 36.71]).

Conclusion

Patients with epilepsy reported high levels of social anxiety that have a negative effect on their QoL. Healthcare providers should be able to provide counseling to the patient and their family members. In addition, social support is important to improve their mobility and socialization with friends and neighbors.

## Introduction

The World Health Organization estimates that there are 4.7 million active cases of epilepsy in the Eastern Mediterranean region [1]. There is a general belief that patients with epilepsy (PWE) suffer from depression alone; however, previous studies, mostly conducted in Western countries, have reported high levels of anxiety and its associated disorders in PWE [2,3]. The prevalence of anxiety and its associated disorders reported by different studies range between 11% and 46% [3-5].

In a population-based study from Canada, it was found that, while the prevalence of epilepsy in the general population was low (0.6%), the prevalence of anxiety disorders was two and a half times higher [6]. In another study from South Korea, social anxiety and social interaction anxiety were identified in 49 (21%) and 24 (11%) patients, respectively [7]. A Danish population-based study found that anxiety and depression increased the risk of completed suicides by 12-fold in PWE than in people without epilepsy [8]. Therefore, the identification of comorbid psychiatric disorders should not be limited to depressive disorders and should always include screening for anxiety disorders.

Few studies have been conducted in Saudi Arabia on PWE suffering from anxiety. A study by Alamri et al. found that approximately 16% of the patients met the criteria for moderate or severe anxiety. In addition, it was noticed that epilepsy diagnosis was associated with a higher risk of anxiety but not depression. The study also concluded that higher anxiety and depression scores were significantly correlated with suicidal ideation [9]. Another study from Riyadh found that 19.7% of PWE scored sufficiently high on anxiety disorders to warrant psychiatrist referral and concluded that psychological disorders are common in PWE in Saudi Arabia [10].

Social anxiety, low self-esteem, and depression are important comorbid conditions in PWE and have a negative impact on patients’ quality of life (QoL) [11,12]. A United Arab Emirates-based study on 160 adult patients with anxiety showed a significant positive correlation with health-related QoL [12]. In addition, sociocultural differences, a dysfunctional family, and a lack of knowledge about epilepsy tended to be associated with social anxiety [7]. In Saudi Arabia, research on social anxiety in PWE remains underrecognized [13,14]. The Saudi public is only aware of the somatic manifestations of the disease, and there is a deficiency in knowledge and awareness of the psychosocial manifestations of epilepsy [13]. In addition, anxiety disorders may interfere with the management of epilepsy, resulting in poor QoL [15-17]. Hence, it is important to identify social anxiety and educate physicians and the public regarding the psychosocial implications of epilepsy. The objective of this study was to measure the prevalence of social anxiety disorder and its association with QoL in PWE at King Khalid University Hospital (KKUH), Riyadh.

## Materials and methods

This was a cross-sectional study conducted from February 2020 to January 2021. The initial plan was to conduct face-to-face interviews; however, due to the COVID-19 outbreak and closure of clinics, we decided to conduct online data collection. A list of 1,033 patients along with their contact numbers was acquired from the Epilepsy Clinic, Department of Neurology, at King Khalid University Hospital (KKUH). Patients aged 18 years and above and having a confirmed diagnosis of epilepsy for more than a year were invited to participate in the study, whereas those who were physically handicapped or mentally impaired were not included in the study. 

The study protocol was reviewed and approved by the Institutional Review Board (IRB) of King Saud University Medical City (No. E-20-5419). The study followed the principles outlined in the Declaration of Helsinki. All participants were asked to read and sign a consent form before the interviews. All data were confidentially treated and utilized only for research purposes after assigning a unique identification number to each participant. No incentives or rewards were provided to the participants. 

In order to explore the association between QoL and social anxiety, assuming a type-I error of 0.05, type-II error of 0.20 (power of 0.80), and a 25% difference in the QoL between those with high and low levels of social anxiety, we required 450 participants. However, due to a poor response rate of around 25%, only 246 of 1,033 patients agreed to answer the online questionnaire.

The data were collected using a self-administered online questionnaire using Google Forms. The researchers contacted the patient via telephone to explain the purpose of the study and obtain consent. Only one participant was included from each family. The researcher asked each participant about their preferred method of receiving the questionnaire (WhatsApp or SMS) and sent a message containing a link to the questionnaire accordingly. For any queries, the participants were directed to a senior researcher.

The questionnaire

The questionnaire comprised five sections, namely, sociodemographic questions (including adverse life events), epilepsy-related information, the Liebowitz social anxiety scale (LSAS), and the European quality-of-life 5-dimensions 3-levels (EQ-5D-3L) scale, and questions on social support. Social support was measured by two questions: the number of people whom you can talk to and the number of people available to help when needed. Information on any adverse life events during the last year was also collected. Adverse life events included death among immediate family members, divorce, loss of job, financial breakdown, accident, etc. 

LSAS

The LSAS measures social anxiety among general and specific populations in everyday situations. The scale measures the range of social interactions and performance situations covered under two sections, one measuring how anxious or fearful one feels while confronting a specific situation, and the second part measuring how often one tends to avoid the situation (avoidance behavior). If a situation is not experienced by the participant, it has to be imagined and answered accordingly. Both sections comprise 24 items, each answered using a Likert scale, ranging from none or never (marked as 0) to usually or severe (marked as 3). The scale was translated into Arabic and previously used in a study conducted in Oman [18]. Cronbach’s Alpha value for reliability was 0.74.

EQ-5D-3L

The EQ-5D-3L is a standardized measure of health originally developed in English and subsequently validated in different languages, including Arabic. The instrument measures QoL in five dimensions: mobility, self-care, usual activities, pain/discomfort, anxiety, and depression. Each dimension has three levels: no problems, some problems, and extreme problems (coded as 1-3). The respondent is asked to indicate their health by choosing the most appropriate statement in each of the five dimensions. Previous studies from Saudi Arabia have utilized the EQ-5D-3L to measure QoL in patients with chronic diseases [19]. Cronbach’s alpha for the reliability analysis was 0.77.

Statistical analysis

The data were analyzed using Statistical Package for Social Sciences®️ (SPSS, version 21; IBM Corp., Armonk, NY, USA). Descriptive analysis was conducted to measure the means and standard deviations for continuous variables and frequency percentages for categorical variables, respectively. Pearson chi-square and p-values were calculated for categorical variables to measure the association between social anxiety and QoL domains. Student’s t-test was used to measure the difference between continuous variables. Following univariate analysis, independent multivariate binary logistic regression was conducted to measure the association between social anxiety and each of the five dimensions of the EQ-5D-3L. Associations that were significant in the univariate analysis or caused ≥10% difference in the estimates were retained in the final model. Each association was adjusted for potential confounders, which included age, sex, income, adverse life events and social support, time since epilepsy diagnosed, and frequency of seizures. All plausible interactions were confirmed before the development of the final model. The Hosmer-Lemeshow test was utilized to predict the model’s goodness of fit. The level of significance was set at p<0.05.

## Results

Participant characteristics

This study included 246 PWE. Their sociodemographic characteristics are presented in Table 1. The participants were aged 18-66 years (mean age: 33.2±12.8 years). Female patients participated more in number as compared to male patients (61% vs. 39%). However, no significant differences were observed between male and female patients in terms of age, education, income, and marital status. The majority of male patients (80%) were working or were students, whereas most of the female patients (60%) were housewives. A significant percentage (36.2%) of patients had suffered from a life event in the previous year. Social support was inadequate as a majority of patients mentioned that only a few people are available with whom they could talk and count on. The majority also mentioned that it was difficult to get help from people around, especially the neighbors.

**Table 1:** Sociodemographic characteristics of patients with epilepsy in a tertiary care hospital in Riyadh, Saudi Arabia (n=246)

Variables		N (%)
Age (Mean± SD)		33.2 (±12.8)
Gender	Male	96 (39)
	Female	150 (61)
Marital status	Single	129 (52.4)
	Married	117 (47.6)
Education	High school or above	134 (54.5)
	Less than high school	112 (45.5)
Occupation	Student	56 (22.8)
	Employed	86 (35)
	Unemployed	104 (42.3)
Family Income (SAR)	>20,000	30 (12.2)
	10,000-20,000	78 (31.7)
	<10,000	138 (56.1)
Any major life event (during the last one year)	Death among close family	42 (17.1)
	Major accident	26 (10.6)
	Divorce	7 (2.8)
	Lost job	14 (5.7)
	None	157 (63.8)
Number of people to talk to and count on	None	16 (6.5)
	1-2	130 (52.8)
	3-4	69 (28)
	≥5	31 (12.6)
Can get practical help from people around (neighbors, friends, etc.)	Very difficult	48 (19.5)
	Difficult	33 (13.4)
	Possible	91 (37)
	Easy	50 (20.3)
	Very easy	24 (9.8)

Clinical features of epilepsy

Table 2 shows that more than 50% of patients had been suffering from epilepsy for more than 10 years. However, only 3.3% (n=8) suffered from seizures on a daily basis. One-fourth (24%) of patients mentioned a family history of epilepsy.

**Table 2:** Clinical features of patients with epilepsy in a tertiary care hospital in Riyadh, Saudi Arabia (N=246)

Characteristics	n (%)
Epilepsy since when (in years)	
1-2	10 (4.1)
3-5	19 (7.7)
>5-<10	86 (35)
≥10	131 (53.3)
Frequency of seizures	
Daily	8 (3.3)
Weekly	14 (5.7)
Monthly	55 (22.4)
Yearly	169 (68.7)
Taking anti-epileptic treatment	
Yes	237 (96.3)
No	9 (1.7)
Past history of any other mental illness	
Yes	30 (12.2)
No	216 (87.8)
Family history of epilepsy	
Yes	59 (24)
No	187 (76)

LSAS scores and QoL scale dimensions

Table 3 shows the descriptive frequencies for the social anxiety scale and the QoL dimensions for PWE. The mean (± standard deviation) scores for the total LSAS, anxiety, and avoidance subscales were 42.2 (±30.7), 21.1 (±17.1), and 21.2 (±17.4), respectively. Approximately 23% of PWE reported mild/ moderate anxiety levels, whereas 15% reported severe/very severe anxiety. No significant differences were observed in the scores between males and females. The majority of patients reported normal QoL scores for each of the dimensions under the QoL scale. No significant sex differences were observed for the QoL dimensions, except for pain and discomfort, which were more frequently reported by female patients.

**Table 3:** Liebowitz social anxiety scores and quality-of-life-scale dimensions as reported by patients with epilepsy LSAS = Liebowitz social anxiety score

Variables		N (%)
LSAS Mean (±SD)		42.4 (±30.7)
Anxiety subscale		21.1 (±17.1)
Avoidance subscale		21.2 (±17.4)
LSAS (categorical)	Normal	154 (62.6)
	Mild	30 (12.2)
	Moderate	26 (10.6)
	Severe	24 (9.8)
	Very Severe	12 (4.9)
Quality of life scale		
Mobility	No problem	201 (81.7)
	Some problem	41 (16.7)
	Confined to bed	4 (1.6)
Self-care	No problem	227 (92.3)
	Some problem	16 (6.5)
	Unable to wash or dress	3 (1.2)
Usual activities	No problem	191 (77.6)
	Some problem	50 (20.3)
	Unable	5 (2)
Pain	No pain or discomfort	127 (51.6)
	Some pain or discomfort	104 (42.3)
	Extreme pain/ discomfort	15 (6.1)
Anxiety	Not anxious or depressed	134 (54.5)
	Moderately anxious/depressed	95 (38.6)
	Severely anxious/depressed	17 (6.9)

Association between social anxiety and QoL domains in PWE

Table 4 shows the multivariate logistic regression model for the association between social anxiety and different dimensions of QoL, namely, mobility, self-care, usual activities, pain or discomfort, and anxiety or depression. Severe social anxiety was significantly associated with restricted mobility (2.65 [1.00, 6.99]), inability to perform usual activities (3.88 [1.61, 9.36]), pain or discomfort (3.21 [1.38, 7.48]), and anxiety and depression (5.77 [2.45, 13.61]). Similarly, a lack of social support (especially from neighbors) was significantly associated with restricted mobility (2.42 [1.12, 5.22]), restricted self-care (3.64 [1.18, 11.17]), inability to perform usual activities (2.86 [1.42, 5.75]), pain or discomfort (2.53 [1.38, 4.66]), and anxiety and depression (1.93 [1.04, 3.57]). Female sex was significantly associated with restricted mobility (2.79 [1.29, 6.03]), whereas low education was associated with restricted self-care (7.38 (1.49, 36.71)] and limited usual activities (2.24 [1.09, 4.58]). All interactions were checked before the development of each association.

**Table 4:** Multivariate logistic regression model showing the association between social anxiety and QoL domains in patients with epilepsy *Association with each dimension was adjusted for age, income, and adverse life events.

Variables		Mobility	Self-care	Usual activities	Pain/discomfort	Anxiety/depression
		Adjusted odds ratio (95% confidence interval)				
Social anxiety	Normal/mild	1.0	1.0	1.0	1.0	1.0
	Moderate/marked	1.12 (0.47,2.65)	0.51 (0.12, 2.11)	1.53 (0.69, 3.41)	1.17 (0.59, 2.29)	3.49 (1.77, 6.88)
	Severe/very severe	2.65 (1.00, 6.99)	1.96 (0.56, 6.81)	3.88 (1.61, 9.36)	3.21 (1.38, 7.48)	5.77 (2.45, 13.61)
Social support	Yes	1.0	1.0	1.0	1.0	1.0
	No	2.42 (1.12, 5.22)	3.64 (1.18, 11.17)	2.86 (1.42, 5.75)	2.53 (1.38, 4.66)	1.93 (1.04, 3.57)
Gender	Male	1.0	1.0	1.0	1.0	1.0
	Female	2.79 (1.29, 6.03)	2.07 (0.68, 6.27)	1.43 (0.71, 2.89)	0.76 (0.43, 1.36)	1.05 (0.58, 1.91)
Education	High school or above	1.0	1.0	1.0	1.0	1.0
	Less than high school	1.56 (0.72, 3.38)	7.38 (1.49, 36.71)	2.24 (1.09, 4.58)	1.55 (0.88, 2.73)	1.37 (0.78, 2.45)

## Discussion

Our results identified that social anxiety is common among PWE, and factors such as social support, sex, and education were all significantly associated with QoL in PWE. In fact, our results found a higher number of patients suffering from social anxiety as compared to previous studies [2,20]. Social anxiety disorders are more common than depression among PWE; in fact, high levels of anxiety may lead to depression [17,21]. Hence, it is important to identify the social anxiety to optimize management and improve the QoL of PWE. It is important to realize that, in addition to the general anxiety associated with having epilepsy, the extreme fear of having a seizure affects cognition and triggers avoidance behavior in almost all situations. Social anxiety triggers excessive fear of being seen by others during a seizure to the extent that it affects normal daily life, leading to anti-social personality and poor QoL [17]. The pathophysiology of the association between epilepsy and anxiety is bidirectional [5,17]. In particular, stressful situations can trigger seizures, which in turn leads to more stress, thus amplifying the effect of a seizure. In addition, the involvement of specific parts of the brain, such as the limbic system, explains the association between stress and seizure [17]. 

Several studies have been conducted on social anxiety among the general population. The reason why social anxiety is more significant for PWE is due to the extreme fear of having a seizure in public and feeling humiliated and judged, which prevents the patients from carrying out their daily activities [22]. As a result, PWEs begin to avoid social interactions, and their mobility becomes restricted. In addition, previous studies from Saudi Arabia have reported a negative attitude toward PWE [13], thus reflecting the general public’s mindset; hence, it is important to recognize and provide counseling to such patients so that their QoL improves. Hence, it is important to educate parents, caregivers, peers, and teachers to help patients overcome the fear and apprehension associated with experiencing a seizure. Additionally, it is important to educate the general population on how to handle seizure episodes, as it is important to raise awareness of seizures in order to establish a community where the general population views epilepsy as a normal medical condition.

The coexistence of psychiatric co-morbidities in PWE, emphasizes the appropriate management for both anxiety and depression, as well as epilepsy [23,24]. The coexistence of depression is natural for individuals with limited social interaction, which leads to loneliness and further depression. We found that severe social anxiety was three times more likely to be associated with severe pain. Animal studies further extended on human beings have confirmed that strong emotional responses affect pain perception [25].

Recently, several studies have examined the positive role of social support in improving the physical and mental health of PWE [26,27]. However, the role of neighbors in improving the QoL of PWE has not yet been explored. We found that support from family members and neighbors can help the patients carry out their daily activities, improving mobility as well as their mental health, and decreasing their pain threshold (odds ratio: 2.00-3.5). Neighbors' support can play a special positive role in improving the QoL of PWE because of the close communication, long relationship, and trust they have for each other [28]. Saudi Arabia has a strong supportive family system, where usually the neighbors are either close family or become like family members. Hence, the patients and families can benefit from this opportunity. In addition, since the majority of Saudi Arabia's population was Muslim, we considered neighborly help, as Islamic teachings encourage good behavior and assisting one's neighbors. Families with PWE may try to establish social groups consisting of neighbors to improve the patient’s social interactions and social circle. This approach may have a positive impact on the QoL and overall mental health of PWE. Longitudinal studies can help in observing and comparing the long-term impact of involving neighbors in the management of PWE between the Middle Eastern and Western cultures. Similar to our study, previous studies have also reported that females show psychiatric comorbidities more frequently than males [24]. However, it is important to educate the public about the effects of social anxiety on both sexes. Although age was not independently associated with QoL domains for PWE, in general, younger age people are found to be more anxious and socially conscious than older aged, which may explain the higher mean scores among young patients. The significant association between education and self-care and usual activities indicates that education not only helps patients take care of themselves but also in performing their daily tasks [29]. Moreover, it has been observed that educated patients have better compliance with their medication adherence and tend to be updated regarding recent research and management techniques. Although we do not have the required data, it is very likely that educated patients are able to access information from different sources that help them improve their QoL.

The major strength of our study is the utilization of validated and specific scales (the LSAS, EQ-5D-3L, and social support scale). There are limitations to this study, such as the fact that patients were recruited from one tertiary care hospital; hence, the results cannot be generalized to patients elsewhere. Furthermore, it is noteworthy that we were unable to account for the predominant seizure type, as a significant proportion of patients did not provide responses to this specific inquiry. The cross-sectional study design did not establish a temporal association between social anxiety and QoL. However, we recommend further research to confirm the relationship between social anxiety and social support in diverse populations, affecting the QoL. Due to the pandemic, we could not conduct face-to-face interviews and had to make telephone contact; hence, information bias may have affected the results. The refusal rate was high leading to selection bias. Some participants did not have the required app to access the questionnaire; hence, they were not able to participate.

## Conclusions

Both social anxiety and lack of social support are independent predictors for poor QoL in PWE. Social anxiety not only restricts mobility and usual activities but also increases general anxiety and depression in PWE. Identification and counseling for social anxiety in PWE is an essential part of management. In addition, improving social support by establishing a strong neighborhood network can help improve the QoL of PWE. It is recommended that both families and the neighborhood be educated and involved in the management of PWE. Furthermore, it is recommended that physicians maintain a heightened level of awareness and use a validated office tool to screen for social anxiety in PWE.

## References

[1] World Health Organization. Regional Office for the Eastern Mediterranean (2010). Epilepsy in the WHO Eastern Mediterranean Region: Bridging the Gap. https://applications.emro.who.int/dsaf/dsa1039.pdf.

[2] Fiest KM, Dykeman J, Patten SB (2013). Depression in epilepsy: a systematic review and meta-analysis. Neurology.

[3] Beyenburg S, Mitchell AJ, Schmidt D, Elger CE, Reuber M (2005). Anxiety in patients with epilepsy: systematic review and suggestions for clinical management. Epilepsy Behav.

[4] Munger Clary HM, Snively BM, Hamberger MJ (2018). Anxiety is common and independently associated with clinical features of epilepsy. Epilepsy Behav.

[5] Kwon OY, Park SP (2014). Depression and anxiety in people with epilepsy. J Clin Neurol.

[6] Tellez-Zenteno JF, Patten SB, Jetté N, Williams J, Wiebe S (2007). Psychiatric comorbidity in epilepsy: a population-based analysis. Epilepsia.

[7] Han SH, Kim KT, Ryu HU (2019). Factors associated with social anxiety in South Korean adults with epilepsy. Epilepsy Behav.

[8] Christensen J, Vestergaard M, Mortensen PB, Sidenius P, Agerbo E (2007). Epilepsy and risk of suicide: a population-based case-control study. Lancet Neurol.

[9] Alamri Y, Al-Busaidi IS (2015). Anxiety and depression in Saudi patients with epilepsy. Epilepsy Behav.

[10] Shariff EM, Sinha S, Samman SK, ElBakri NK, Siddiqui KA, Mahmoud AA (2013). Depression and anxiety in parents of children with epilepsy. Are fathers involved?. Neurosciences (Riyadh).

[11] Kutlu Ae, GÖKÇE Gke, BÜYÜKburgaz l, Selekler M, KomŞUoğLu S (2013). Self-esteem, social phobia and depression status in patients with epilepsy. Nöro Psikiyatri Arşivi.

[12] Alsaadi T, Kassie S, El Hammasi K (2017). Potential factors impacting health-related quality of life among patients with epilepsy: results from the United Arab Emirates. Seizure.

[13] Alaqeel A, Sabbagh AJ (2013). Epilepsy; what do Saudi's living in Riyadh know?. Seizure.

[14] Alkahil HB, Falatah YM, Abdulbaki MM (2017). Awareness about epilepsy in children among schoolteachers in Riyadh, KSA. Int J Adv Res.

[15] Petrovski S, Szoeke CE, Jones NC, Salzberg MR, Sheffield LJ, Huggins RM, O'Brien TJ (2010). Neuropsychiatric symptomatology predicts seizure recurrence in newly treated patients. Neurology.

[16] Kanner AM, Byrne R, Chicharro A, Wuu J, Frey M (2009). A lifetime psychiatric history predicts a worse seizure outcome following temporal lobectomy. Neurology.

[17] Hingray C, McGonigal A, Kotwas I, Micoulaud-Franchi JA (2019). The relationship between epilepsy and anxiety disorders. Curr Psychiatry Rep.

[18] Al-Sharbati M, Al-Adawi S, Petrini K (2012). Two-phase survey to determine social anxiety and gender differences in Omani adolescents. Asia Pac Psychiatry.

[19] Algahtani HA, Shirah BH, Alzahrani FA, Abobaker HA, Alghanaim NA, Manlangit JS Jr (2017). Quality of life among multiple sclerosis patients in Saudi Arabia. Neurosciences (Riyadh).

[20] Altwijri RM, Aljohani MS, Alshammari HK (2021). Quality of life among epileptic patients in Qassim Region, KSA. Neurosciences (Riyadh).

[21] Wiglusz MS, Landowski J, Cubała WJ (2018). Prevalence of anxiety disorders in epilepsy. Epilepsy Behav.

[22] Blixen C, Ogede D, Briggs F (2020). Correlates of stigma in people with epilepsy. J Clin Neurol.

[23] Altintas E, Yerdelen VD, Taskintuna N (2015). Social anxiety level in adult patients with epilepsy and their first-degree cohabiting relatives. J Neuropsychiatry Clin Neurosci.

[24] Kimiskidis VK, Triantafyllou NI, Kararizou E (2007). Depression and anxiety in epilepsy: the association with demographic and seizure-related variables. Ann Gen Psychiatry.

[25] Rhudy JL, Meagher MW (2000). Fear and anxiety: divergent effects on human pain thresholds. Pain.

[26] Charyton C, Elliott JO, Lu B, Moore JL (2009). The impact of social support on health related quality of life in persons with epilepsy. Epilepsy Behav.

[27] Gülpek D, Bolat E, Mete L, Arici S, Celebisoy M (2011). Psychiatric comorbidity, quality of life and social support in epileptic patients. Nord J Psychiatry.

[28] Guo W, Wu J, Wang W, Guan B, Snape D, Baker GA, Jacoby A (2012). The stigma of people with epilepsy is demonstrated at the internalized, interpersonal and institutional levels in a specific sociocultural context: findings from an ethnographic study in rural China. Epilepsy Behav.

[29] Dash D, Sebastian TM, Aggarwal M, Tripathi M (2015). Impact of health education on drug adherence and self-care in people with epilepsy with low education. Epilepsy Behav.

